# OLDER VETERANS’ PREFERENCES FOR FORMAT AND DESIGN OF EDUCATIONAL MENTAL HEALTH BROCHURES

**DOI:** 10.1093/geroni/igac059.2866

**Published:** 2022-12-20

**Authors:** Ruifeng Cui, Armando Rotondi, Alaa Shalaby, Judith Callan

**Affiliations:** VA Pittsburgh Healthcare System, Morgantown, West Virginia, United States; VA Pittsburgh Health Care, Pittsburgh, Pennsylvania, United States; VA Pittsburgh Healthcare System, Pittsburgh, Pennsylvania, United States; VA Pittsburgh Health Care, Pittsburgh, Pennsylvania, United States

## Abstract

Older Veterans may have lower mental health literacy and education than younger Veterans. The Veterans Affairs Healthcare System (VA) has created many educational brochures on mental health to facilitate service utilization by Veterans, however few studies have solicited Veteran stakeholder feedback pertaining to their design and effectiveness. The present qualitative pilot study aimed to develop a brief general mental health educational brochure for older Veterans seen in VA medical settings. The brochure aims to provide information on mental health symptoms and VA mental health resources. Participants (Nf5, mean age=67.2) were older adult White male Veterans. An iterative design process was used to develop and refine the educational brochure which consisted of qualitative interviews with Veteran stakeholders on their mental health needs and their feedback on the brochure. The brochure was then modified based on prior Veteran feedback before being tested with subsequent Veteran participants. The brochure received high ratings on clarity (8.8/10), conciseness (8.6/10), and importance (8.6/10). Qualitative interviews revealed that Veterans appreciated the simplistic presentation of the content (“It’s very informative for being so simple, it caught my attention”), the generality of the content (“it is encouraging because it is a broad approach to a spectrum of mental health concerns”), and contact number content (“Direct lines, that’s really nice”). These findings support the use of user-centered design methods to develop educational materials. The findings indicated that a brief educational mental health print brochure was an acceptable method to promote mental health literacy and use of VA mental health services.

